# Parasagittal Interlaminar and Transforaminal Epidural Steroid Injections for Radicular Low Back Pain; Which is More Comfortable?

**DOI:** 10.4274/TJAR.2023.231470

**Published:** 2023-12-27

**Authors:** Gevher Rabia Genç Perdecioğlu, Gökhan Yıldız, Ömer Taylan Akkaya, Ezgi Can, Damla Yürük

**Affiliations:** 1 Ankara Etlik City Hospital, Clinic of Algology, Ankara, Turkey

**Keywords:** Algology, epidural steroid injection, pain, parasagittal interlaminar, transforaminal epidural

## Abstract

**Objective:**

This study aimed to compare parasagittal interlaminar (PS) and transforaminal (TF) epidural steroid injections for unilateral L5 and S1 radicular lower back pain in terms of patient comfort, efficacy, safety, contrast enhancement, and radiation exposure.

**Methods:**

This was a prospective randomized single-blind study. A total of 59 participants were included in this study. The visual analog scale (VAS) and Oswestry Disability Index (ODI) were obtained. A comfort questionnaire was administered to all participants. The total fluoroscopy time and contrast distribution levels were recorded.

**Results:**

Pre- and post-treatment VAS scores were similar between the groups. The ODI scores increased in favor of the PS group at week 2 (*P* < 0.041); however, there was no difference between the two groups at other times. The VAS and ODI scores improved significantly with treatment in both the groups (*P* < 0.001). Total fluoroscopy time was shorter in the PS group (*P* < 0.001). PS application was more comfortable (*P* < 0.001). While no complications were observed in the PS group, three complications occurred in the TF group. Anterior epidural contrast spread to three or more levels was observed in 57% of the participants in the PS group, whereas no spread to more than two levels was observed in the TF group.

**Conclusion:**

The PS epidural approach is superior to the TF approach in terms of a low incidence of side effects, less radiation exposure, better patient comfort, higher epidural contrast spread, and single-level needle access.

**Main Points**

• In our study, parasagittal epidural steroid injection was superior to the transforaminal method for the treatment of radicular low back pain at week 2 and similar efficacy at week 4.

• Total radiation dose, side effects, and patient comfort were superior to transforaminal.

• Our aim was to analyze the advantages and disadvantages of methods with similar efficacy. The parasagittal approach seems to be more useful than the transforaminal approach.

## Introduction

One of the most common causes of chronic low back pain is a herniated disc.^1^ Radicular is caused by inflammation of herniated disc material in the epidural space. It is treated with epidural steroids, especially dexamethasone.^2,3,4^ Epidural steroid injections can be performed using caudal, transforaminal, midline, and parasagittal interlaminar approaches.

Previous studies have compared these methods in terms of treatment efficacy, contrast spread, and side effects. Many reports suggest that treatment efficacy is superior for PS and TF interventions than for caudal and midline interlaminar epidural approaches.^5,6,7,8^

However, there is no clear answer as to which of these two methods is preferable. The effectiveness of the parasagittal interlaminar (PS) and transforaminal (TF) approaches has generally been found to be similar.^6,9,10,11^ In terms of safety, in contrast to the benign nature of the PS approach, the TF approach appears to have a higher risk of complications because of its proximity to the radicular medullary artery and nerve root.^8,10,12,13,14,15^ Authors have different opinions on contrast distribution and fluoroscopy time.^6,7,8^

In this study, we compared the TF and PS methods for radicular low back pain due to L4-L5 and L5-S1 posterolateral disc herniation. We aimed to determine the superiority of these two techniques in terms of safety, total radiation exposure, patient comfort, and contrast enhancement.

## Methods

### Study Design and Population

This was a prospective, randomized, controlled clinical trial. Ethics Committee approval was obtained from the University of Health Sciences Turkey, Dışkapı Yıldırım Beyazıt Training and Research Hospital Ethics Committee, and we are affiliated with and registered in Clinical Research (date: 07.03.2022, approval no: 132/10, Clinical Trial Number: NCT05551676).

Between August 2022 and January 2023, 123 patients with unilateral radicular low back pain were assessed. Of the 123 patients who met the inclusion criteria, 59 were included in the study. The participants underwent treatment in the Department of Algology. The inclusion criteria were as follows: 1) age 20-60 years; 2) radiologically proven L4-L5 and L5-S1 protruded/extruded discs with radicular symptoms; and 3) >3 months of pain that did not respond to conservative treatment. The exclusion criteria were as follows: 1) migrated disc or spinal stenosis (anteroposterior spinal canal diameter less than 12 mm on lumbar magnetic resonance images); 2) previous lumbar surgery or algological procedure; 3) indication for emergency surgery for discopathy; 4) malignancy, pregnancy, or other rheumatological/neurological diseases; and 5) no contrast spread to the anterior epidural space and target nerve roots during the procedure.

We used a computer-assisted randomization program to categorize the patients into two groups: the PS group was assigned number 1, and the TF group was assigned number 2. The sample size was based on the primary outcomes and calculations using G*Power 3.1.9.4 software, with an effect size of 0.617, α=0.05, and power (1-β) =0.80.^16,17^ A total of 40 subjects were included in each group. Kaur’s third-month visual analog scale (VAS) scores [mean and standard deviation (SD)] were obtained for this analysis.^7^ A literature search was performed using PubMed from the National Library of Medicine.

**Figure 1:**
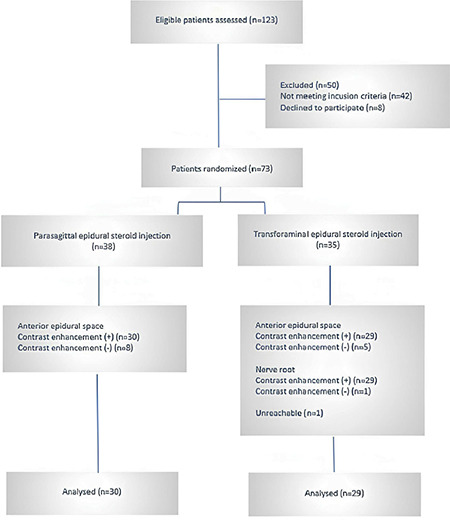
Flow chart diagram.

The study design is illustrated in Figure 1.

### Intervention

Both procedures were performed under fluoroscopic guidance without sedation. To avoid dural puncture, needle distance was controlled in the lateral view using C-arm fluoroscopy. During the procedure, 4 mL of the contrast agent was administered. The number of vertebral levels spread by the contrast agent in the anterior epidural space was also recorded. As all participants had bi-level disc herniation, the procedure was performed at the clinically most prominent root level in the PS group, and at both root levels in the TF group.

### Transforaminal Epidural Approach

A 22-gauge, 3.5-inch blunt-tip atraumatic needle was used. The L4-L5 and L5-S1 intervertebral foraminas were approached using the subpedicular (safe triangle) technique. We injected 2 mL of contrast medium at each level to determine epidural spread. We administered 4 mL of drug into each nerve root: two mL of dexamethasone 21-phosphate, one cc of 0.5% bupivacaine HCl, and one cc of saline.

### Parasagittal Interlaminar Epidural Approach

**Figure 2:**
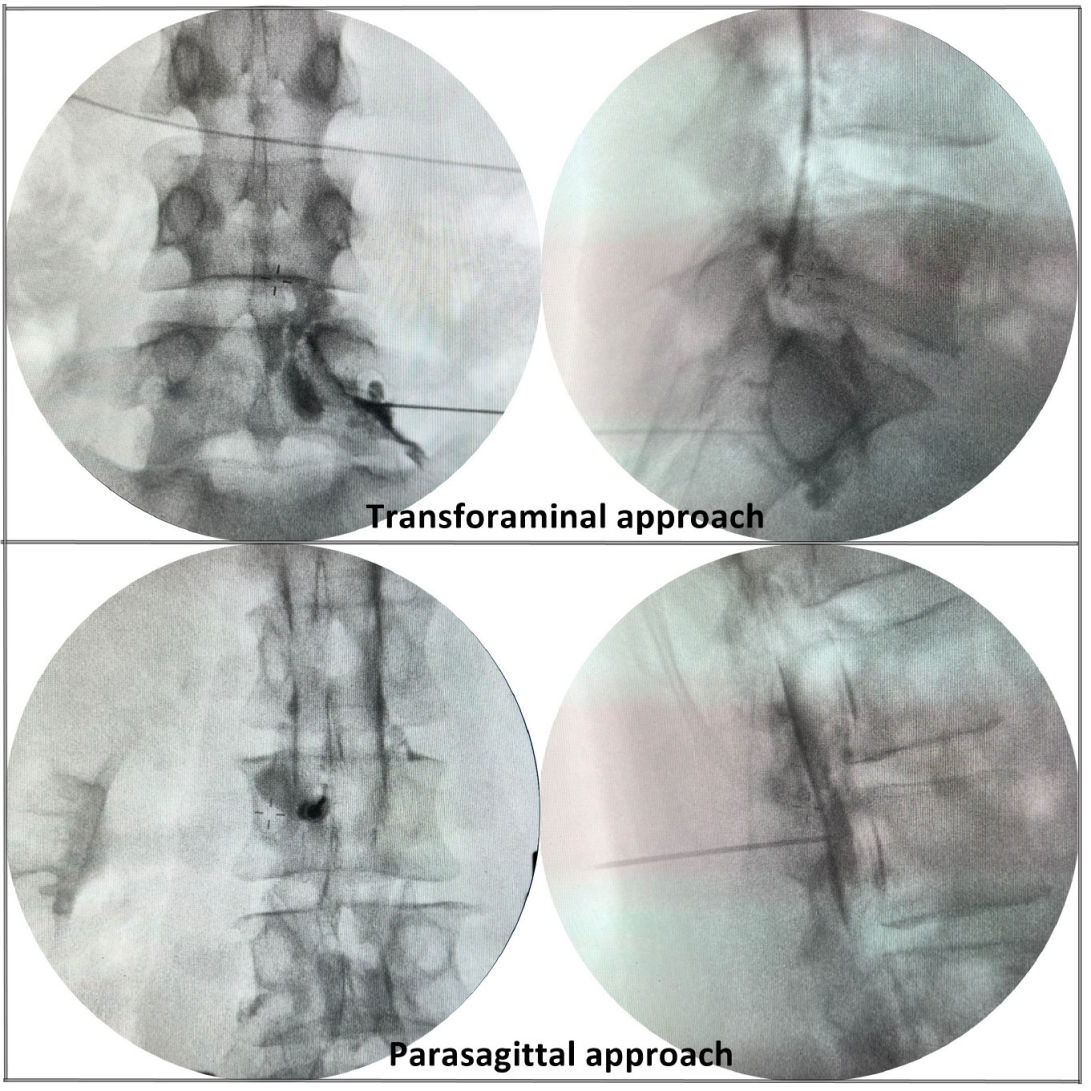
Upper left image: Anteroposterior view in transforaminal approach, Upper right image: Lateral view in transforaminal approach, Bottom left image: Anteroposterior view in parasagittal approach, Bottom right image: Lateral view in parasagittal approach.

An 18 gauge, 3.5 inc Tuohy needle was used. The entry point was approximately 1.5 cm lateral to the midline on the side of the painful lower extremity in the L4-L5 or L5-S1 interlaminar space. Epidural space was obtained using a loss-of-resistance technique. After entering the epidural space, 4 mL contrast medium was administered. Spread into the anterior epidural space was observed and vertebral levels were noted. Four mL of dexamethasone 21-phosphate, two mL of 0.5% bupivacaine HCl and two cc of saline were injected, resulting in a total of eight mL of the drug. The contrast distribution is shown in Figure 2.

### Outcome Measures

VAS, Oswestry Disability Index (ODI), and comfort questionnaires were administered to all the patients. Total fluoroscopy time, extent of contrast spread into the anterior epidural space, and adverse events were recorded. The primary outcome measure was improvement in pain intensity. We asked the patients to report their VAS before and 2-4 weeks after treatment. Secondary outcomes were between-group differences in functionality improvement, fluoroscopy time, patient comfort, and side effects or complications. We assessed the improvement in functionality using the ODI score. The ODI is a patient-completed questionnaire that measures the functioning of patients with low back pain. The time at which the fluoroscopy device was active during the procedure was also recorded. We asked the patients to complete a comfort questionnaire after the procedure and to answer how they felt during the procedure using one of three options: comfortable, moderate, and uncomfortable. While answering this question, we asked them to rate the length of time they spent in the operating theatre and the pain they felt during and after the procedure. We monitored the patients for side effects during and after the procedure, and recorded their occurrence.

### Statistical Analysis

All analyses were performed using Jamovi Project (2022, Jamovi version 2.3) (computer software). The results of this study are expressed as frequencies and percentages. Normality analysis was performed using the Shapiro-Wilk test, skewness kurtosis, and histograms. Normally distributed variables are presented as means and SDs. Categorical variables were compared using the chi-squared test. Independent samples t-tests and Mann-Whitney U tests were used to compare numerical dependent variables between the groups. Repeated measures were analyzed using Friedman’s test with Bonferroni correction for multiple t-tests. Statistical significance was set at *P* < 0.05.

## Results

Fifty-nine patients completed the third month of the follow-up. There was no difference in age or sex between the two groups (*P* > 0.05; independent samples t-test, chi-square test). We compared the VAS and ODI scores before and 2-4 weeks after the procedure (independent samples t-test, paired samples test, and Friedman test).

Fluoroscopy time and comfort scale scores were compared between the groups (independent samples t-test, Fisher’s exact test, continuity correction, Pearson’s chi-squared test, and Mann-Whitney U test) (Table 1).

There was no difference in the pre- and post-treatment VAS scores between the two groups (Table 1). When analyzed within each group, the decrease in the VAS scores over time was significant (*P *< 0.001 for both groups). When the change between time points was analyzed, the change between baseline two weeks and baseline four weeks was significant in both groups (Bonferroni correction; *P *< 0.001, both). There were no differences between the measurements at two and four weeks after treatment in either group (Table 2).

There was no difference in the ODI scores between the groups at baseline; a statistically significant decrease was observed in the PS group compared with the TF group in the second week (mean rank PS: 25.52, TF: 34.64,* P*=0.041) (Table 1). No differences were found between ODI measurements in the fourth week. When the change in ODI scores was analyzed over time, a significant decrease from baseline was observed in both groups (*P *< 0.001 for both). When the difference between time points was analyzed, the change in the ODI score between the basal 2 weeks and basal 4 weeks was significant in both groups (Bonferroni correction; *P *< 0.001 for both groups). There was no significant difference between the two- and 4-week scores in either group (Table 2).

Total fluoroscopy time was 15.1±1.93 seconds in the PS group and 49.72±2.78 seconds in the TF group (*P *< 0.001). This duration was more than three times longer in the TF group than that in the PS group. According to the comfort query, 50% of the PS group replied comfortably, 40% moderately, and 10% uncomfortable. In the TF group, 51.7% of participants reported discomfort, 34.4% reported moderate discomfort, and 13.7% felt comfortable. This difference was statistically significant (*P *< 0.001). We recorded the number of levels of contrast medium that had spread into the anterior epidural space. In the PS group, 57% of the patients had three or more levels of contrast spread, whereas in the TF group, we did not record three levels of contrast enhancement (Table 1).

**Figure 3:**
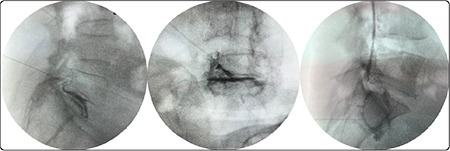
The left and middle images: Contrast enhancement of disc penetration, The right image: Vascular penetration.

No adverse events were observed in the PS group. Three complications occurred in the TF group: one case of disc penetration, one case of vascular penetration, and one patient who experienced transient paralysis for five hours (Figure 3). In the cases of intravascular injection and disc penetration, the procedure was successfully performed by changing the needle position and achieving the desired contrast distribution. The patient with transient paralysis was discharged 24 h after observation. At week 2, four patients in the TF group had increased pain compared to baseline, but by week 4, their pain was relieved.

## Discussion

This study showed that PS and TF epidural steroid injections were successful in treating radicular low back pain due to L4-5 and L5-S1 posterolateral disc herniations. At the end of the first month, both treatments resulted in a 60% reduction in pain intensity and 50% improvement in function. According to our results, the VAS and ODI scores at week 4 were similar in both the groups. However, the ODI score was significantly lower in the PS group at week 2 (*P*=0.041). In the literature, the efficacy of TF ESI and PS epidural approaches has generally been found to be similar.^6,9,10,11^ However, in a meta-analysis comparing the two methods, the PS approach was found to be superior for pain relief, but no difference was found in terms of functionality.^18^ In the results of studies comparing midline, PS and TF approaches are conflicting.^6,19,20^

Epidural steroid injections are the cornerstone of treatment of low back pain caused by herniated discs or spinal stenosis. Injection into the epidural space began in the 1950s, using a caudal approach. Since the 1990s, interlaminar and transforaminal approaches have been used.^8^ However, there is no consensus on which method is preferable.

Epidural approaches have been compared in patients with discogenic radicular low back pain but not in a homogeneous population. In previous studies, the level of disc herniation differed between patient groups. In this study, we evaluated the most common posterolateral protruded and extruded discopathy at the L4-L5 and L5-S1 levels, which had the highest incidence of herniation.^21^

The TF and PS approaches have become popular because of their easier access to the anterior epidural space. Anterior epidural contrast distribution is higher with PS and TF than with midline interlaminar administration,^22^ but there is no consensus on the superiority of these techniques.^4,6,7,11,20,23^ In our study, all patients in the PS group had at least two levels of contrast enhancement in the anterior epidural space, and 57% had three or more levels of contrast enhancement. In the TF group, the contrast remained at the levels we provided, and we did not observe three levels of contrast enhancement in any patient. Given this situation, the wide distribution achieved with a single injection in the PS approach is remarkable.

In our study, fluoroscopy time was much shorter in the PS group. This result was not surprising for this method, which was easier to perform. In contrast, the patient and pain practitioner were exposed to three times more radiation during the TF approach. Previous authors also reported a shorter fluoroscopy time with the PS method compared to the TF method.^20,23^ However, in an article comparing the midline, PS and TF methods, this time was found to be similar for all three methods.^6^

We observed three complications in the TF group: disc penetration, vessel penetration, and transient paralysis. The absence of complications and low radiation exposure due to the shorter fluoroscopy time made the PS method more reliable. Intravascular penetration, spinal cord infarction, paraplegia, permanent paralysis and discitis have been reported with TF epidural steroid injections.^12,13,14,15,18^

To the best of our knowledge, these two methods have not been evaluated in terms of patient comfort. According to the comfort questionnaire, patient satisfaction was four times higher in the PS group than in the TF group.

### Study Limitations

The short follow-up period is the main limitation of this study. In addition, we did not evaluate the analgesics used. However, comparing these two methods in patients with isolated L5 and S1 radiculopathy was an advantage of our study. Therefore, more reliable data were obtained.

## Conclusion

In the treatment of L4-L5 and L5-S1 radiculopathy, the PS epidural approach produced a significantly greater improvement in the ODI scores at two weeks and was at least as effective as TF in reducing pain and improving function. Low adverse events and radiation exposure, improved patient comfort, and wide contrast distribution with a single-level procedure make the PS epidural approach preferable.

## Figures and Tables

**Table 1:**
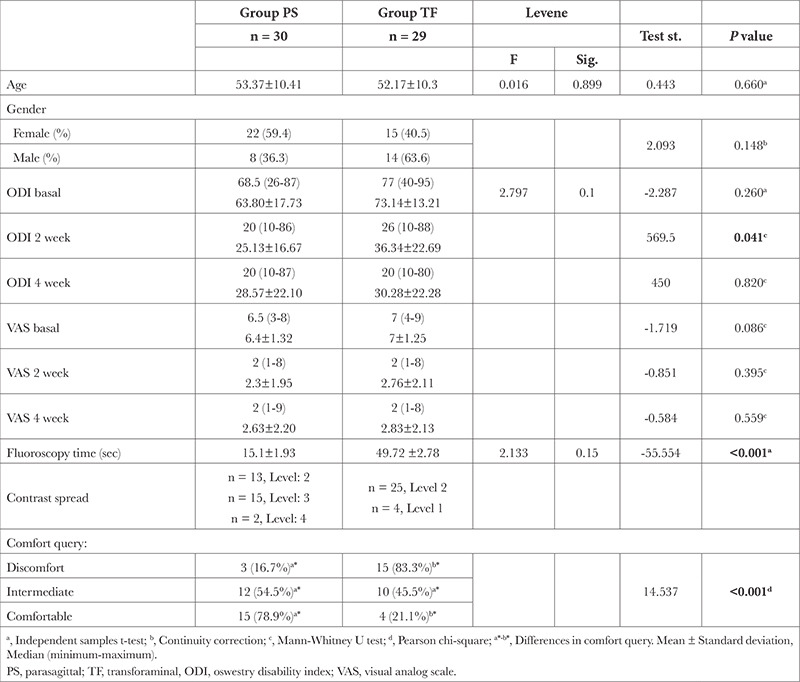
Demographic Data and Group Comparison

**Table 2:**
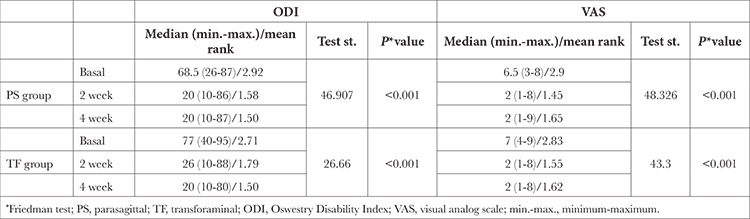
ODI and VAS Scores Over Time
